# The role of perceived air pollution and health risk perception in health symptoms and disease: a population-based study combined with modelled levels of PM_10_

**DOI:** 10.1007/s00420-018-1303-x

**Published:** 2018-03-31

**Authors:** Kati Orru, Steven Nordin, Hedi Harzia, Hans Orru

**Affiliations:** 1 Department of Psychology, Umeå University, Umeå, Sweden; 2 Institute of Social Studies, Tartu University, Tartu, Estonia; 3 Estonian Health Board, Tallinn, Estonia; 4 Institute of Family Medicine and Public Health, Tartu University, Tartu, Estonia; 5 Department of Public Health and Clinical Medicine, Umeå University, Umeå, Sweden

**Keywords:** Perceived pollution, Epidemiology, Path analysis, Air pollution modelling, Questionnaire survey

## Abstract

**Purpose:**

Adverse health impact of air pollution on health may not only be associated with the level of exposure, but rather mediated by perception of the pollution and by top-down processing (e.g. beliefs of the exposure being hazardous), especially in areas with relatively low levels of pollutants. The aim of this study was to test a model that describes interrelations between air pollution (particles < 10 ${\upmu }$m, PM_10_), perceived pollution, health risk perception, health symptoms and diseases.

**Methods:**

A population-based questionnaire study was conducted among 1000 Estonian residents (sample was stratified by age, sex, and geographical location) about health risk perception and coping. The PM_10_ levels were modelled in 1 × 1 km grids using a Eulerian air quality dispersion model. Respondents were ascribed their annual mean PM_10_ exposure according to their home address. Path analysis was performed to test the validity of the model.

**Results:**

The data refute the model proposing that exposure level significantly influences symptoms and disease. Instead, the perceived exposure influences symptoms and the effect of perceived exposure on disease is mediated by health risk perception. This relationship is more pronounced in large cities compared to smaller towns or rural areas.

**Conclusions:**

Perceived pollution and health risk perception, in particular in large cities, play important roles in understanding and predicting environmentally induced symptoms and diseases at relatively low levels of air pollution.

## Introduction

There is strong epidemiological evidence for relationships between short- and long-term exposure to particulates and cardiopulmonary mortality, hospitalization and respiratory disease (e.g. asthma, chronic bronchitis, rhinitis) (WHO 2013). Recent evidence relates air pollution to diabetes (Thiering and Heinrich 2015), rheumatic diseases (Sun et al. 2016), cognitive functioning (Clifford et al. 2016) and neurodegenerative diseases (Oudin et al. 2016; Xu et al. 2016). The main mechanism behind the development of diabetes and rheumatoid arthritis is oxidative stress and initial inflammation. Experimental evidence suggests air pollution increasing systemic insulin resistance (Brook et al. 2013) and oxidative stress in the lungs may be an intermediate step between exposure and health effects (Haberzettl et al. 2016). Hence, persistent amplified chronic inflammation together with production of cytokines (e.g. IL-1, IL-6) may lead to rheumatoid arthritis (Ruiz-Esquide and Sanmartí 2012; Ying et al. 2015). Moreover, air pollution may induce oxidative stress also in the brain, similar to that seen in cells elsewhere in the body (Clifford et al. 2016), appeal and accumulate larger deposits of beta-amyloid, as a pathological driver of neurodegeneration.

Apart from direct health effects induced by exposure, the mere perception of pollution may cause health symptoms as a protective mechanism. If the source is recognised based on its odorous properties and an association of the properties with prior experience is established, the tone of the association (pleasant or unpleasant) will determine whether stress-induced physiological activity in the autonomic nervous system and related brain regions will be lower or higher than normal, respectively (Engen 1991). If the recognised source is perceived as unpleasant it is likely to have a negative impact on health, e.g. evoke annoyance, worry, and disgust as a protective mechanism (Sucker et al. 2008). Apart from olfaction, the trigeminal sensory system plays a critical role in this context since it, in addition to vaporous substances, is activated by particles. Trigeminal chemoreception generates sensations of pungency and irritation, with the primary function of acting as a sentinel of the airways where its reflexively stops inspiration to prevent inhalation of potentially life-threatening substances (Silver 1991). It can be considered as a distinct chemical warning system since substances with strong activation are likely to be potentially harmful. The warning feature is further illustrated by defense reflexes in the body in response to this type of chemical stimulation, such as sweating, coughing, mucus release, tearing, and salivary flow. Among persons with chemical hypersensitivity, even very weak odorous and trigeminal stimuli may, when having a negative association, evoke symptoms ranging from airway to mucosal, skin, gastrointestinal, head-related, cardiac, cognitive and affective symptoms (Andersson et al. 2009).

Another factor that may lead to health symptoms, and possibly also to disease, is worry due to health risk perception, due to the belief that the exposure is hazardous. This top-down mechanism involves higher order neural processing in the brain, which affects emotions, physiology, behaviour and health. Health risk perception involves the individual’s beliefs, attitudes, judgments and feelings. Beliefs about a certain chemical/physical exposure being hazardous (irrespective of actually being hazardous or not), and the worry and stress this evokes, has been shown to contribute to health symptoms (Stenlund et al. 2009; Claeson et al. 2013). This is likely to be explained by mechanisms such as stress-induced inflammation and expectations. Experimental manipulation of health risk perception has shown that belief of exposure being potentially hazardous may generate more health symptoms compared to belief of the exposure having positive health effects (Andersson et al. 2013; Crichton and Petrie 2015). In addition to symptoms, there is evidence that long-term worry and stress are associated with enhanced cardiovascular, endocrinological, immunological and neurovisceral activity that may result in inflammatory and cardiovascular disease and diabetes (Brosschot et al. 2006; Lloyd et al. 2005). In conclusion, perceived air pollution and health risk perception may play important roles in eliciting health symptoms and potentially contribute to disease.

Aspects that may modify risk perception include social status and level of perceived control a person has over one’s wellbeing, and are thereby important predictors of health symptoms. For example, symptoms are higher among persons with low level of education and members of minority groups (Lissåker et al. 2014; Orru et al. 2015). This relationship has been explained by the capacity to cope with risks related to one’s physical or psychological well-being. If a person encounters stressful situations and her belief that her coping capacity is not suitable to handle the situation in an adaptive way, psychological stress might increase the health symptoms, or cause anxiety and fear (Runeson and Norbäck 2013). Deprived populations’ higher perceived risks are related to them suffering worse health effects from air pollution through increased exposure and increased vulnerability to the effects of exposure (Science for Environment Policy 2016). Individuals with low income and education are more likely to afford living space in lower quality neighbourhoods, and their unhealthier lifestyles in addition to pollution levels may contribute to poorer health outcomes (Bilger and Carrieri 2015). Furthermore, people who are healthy may have better possibilities to live in neighbourhoods with better access to green spaces.

The aim of present study was to test a path analytic model that describes the interrelations between physical air pollution (particles < 10 µm), perceived air pollution, health risk perception, health symptoms and diseases in Estonia. According to the model, air pollution leads to perceived pollution, symptoms and disease, perceived pollution leads to health risk perception and symptoms, and health risk perception leads to symptoms and disease. In earlier studies investigating complaints from air pollution (Stenlund et al. 2009; Claeson et al. 2013), an initial model was tested, which included interrelations between air pollution, perceived pollution, health risk perception, annoyance and health symptoms. In Stenlund et al. (2009), annoyance, but not health symptoms, was found to be directly evoked by air pollution (dust and soot), whereas the relation between air pollution and symptoms was mediated by perceived pollution and health risk perception. Another difference between the Stenlund and the present study is that the air pollution levels in the former study were typically high enough to cause explicit concern among residents in that community, which was not the case in the present study. Thus, the present study aimed at testing a model for rather common levels of air pollution. Notably, although the model shows arrows that indicate the most likely direction, the cross-sectional nature of the data does not enable identification of causal effects.

## Materials and methods

### Population and sample selection

The present study was part of a survey on environmental health risk perception and coping, conducted in Estonia in 2015 (Orru et al. 2015). Among the 1.3 million inhabitants in Estonia, a sample of 2207 persons aged 18−75 years, stratified by age, sex, and geographical location in Estonia, was invited to participate (administered by IBP Saar Poll), of which 1000 agreed (45.3% response rate).

### Air pollution exposure modelling

Annual mean concentrations of particulate matter (PM_10_) in 2012 were modelled with the resolution of 1 × 1 km across Estonia using a Eulerian air quality dispersion model that is part of the Airviro Air Quality Management System (SMHI, Sweden; http://airviro.smhi.se). A detailed description of the model is given in the Airviro User Documentation (Airviro 2011). Airviro is a widely used web-based air pollution data management tool that uses data on air emission, level of air pollution and meteorological variables, and has been implemented in several studies (e.g. Orru et al. 2011, 2016a).

We used data on PM_10_ concentrations in Estonia at a spatially disaggregated subnational level. Previous studies in Northern Europe have shown particulate matter, including both coarse (PM_2.5−10_) and fine particles (PM_2.5_), to be a representative indicator of air pollution from regional and local sources such as traffic exhausts, road dust, local heating and industrial sources (Norman and Johansson 2006; Orru et al. 2011). Obtained annual concentrations of PM_10_ in the grid cells were linked with geo-coded survey respondents’ home address in ArcGIS.

### Respondents

Data on PM_10_ emission level at the respondent’s home address were used to categorise the respondent as being exposed to either low (≤ 5.97 ${\upmu }\text{g}/$m^3^), medium (5.98−9.09 ${\upmu }\text{g}/$m^3^) or high (≥ 9.10 ${\upmu }\text{g}/$m^3^) level. Based on this categorisation, each exposure-level group is described in Table 1 with respect to demographics, smoking and walking habits. The three exposure groups did not differ significantly with respect to any of these variables, including the effect of income and the level of urbanity of the living place (Table 1; *α* = 0.05).

**Table 1:** Frequency (%) data on demographics and smoking and walking habits for three groups exposed to different levels of air pollution, and results from group comparisons

	Exposure level				
	Low	Medium	High	Chi^2^	*p* value
Men	148 (33.5)	138 (31.2)	156 (35.3)	1.44	0.49
Married/cohabiting	197 (34.0)	197 (34.0)	186 (32.1)	1.49	0.48
Age (years)				5.38	0.49
18–29	46 (27.5)	57 (34.1)	64 (38.3)		
30–44	94 (33.8)	98 (35.3)	86 (30.9)		
45–64	131 (34.4)	118 (31.0)	132 (34.6)		
≥65	63 (36.2)	57 (32.8)	54 (31.0)		
Highest level of education				4.56	0.34
Elementary school	52 (34.4)	45 (29.8)	54 (35.8)		
High school	182 (33.1)	186 (33.8)	182 (33.1)		
University	97 (33.4)	97 (33.4)	96 (33.1)		
Income group (euros monthly per person in household)				6.83	0.56
≥ 350	95 (33.3)	88 (30.9)	102 (35.8)		
351–450	79 (39.7)	61 (30.7)	59 (29.6)		
451–600	53 (34.4)	54 (35.1)	47 (30.5)		
601–750	39 (32.2)	40 (33.1)	42 (34.7)		
≥750	53 (28.8)	65 (35.3)	66 (35.9)		
Home language				1.48	0.48
Estonian	223 (33.0)	231 (34.2)	221 (32.7)		
Russian/other	111 (34.2)	99 (30.5)	115 (35.4)		
Children in household					
<6 years	51 (38.6)	39 (29.5)	42 (31.8)	1.94	0.38
<18 years	101 (32.1)	109 (34.6)	105 (33.3)	0.61	0.74
Level of urbanity				2.57	0.63
Large city	163 (32.1)	169 (33.3)	175 (34.5)		
Town	76 (37.8)	65 (32.3)	60 (29.9)		
Rural area	95 (32.5)	96 (32.9)	101 (34.6)		
Current smoker	102 (30.7)	118 (35.5)	112 (33.7)	4.49	0.11
Walking (times weekly)				5.7	0.22
≤2	120 (34.1)	124 (35.2)	108 (30.7)		
3–5	112 (35.6)	102 (32.4)	101 (32.1)		
≥6	98 (30.2)	102 (31.4)	125 (38.5)		

Further information on the sample of relevance for the model, including responses to the questions used to represent the factors in the path analysis, is given in Table 2 (perceived air pollution), 3 (health risk perception), 4 (symptoms) and 5 (diseases and perceived health). The three exposure groups differed significantly with respect to perceived exposure to industrial air pollution (higher level of perceived exposure in higher level of air pollution), worry regarding health risks for oneself and family (lowest in the medium exposure group), and prevalence of arthritis (higher in the high exposure group; *α* = 0.05). However, no other significant group differences were found. On the scales ranging from 1 to 5, the average ratings given by the entire sample ranged from 2.09 to 3.19 for perceived air pollution, from 2.29 to 3.45 for health risk perception and from 1.62 to 2.01 for symptoms.

**Table 2:** Mean ratings (entire sample) and response frequencies (%) regarding perceived air pollution for three groups exposed to different levels of air pollution, and results from group comparisons

	Mean	Exposure level				
To what extent are you usually exposed to the following factors^a^		Low	Medium	High	Chi^2^	*p* value
Traffic exhaust^b^	3.19	126 (31.3)	134 (33.3)	143 (35.5)	1.58	0.45
Street dust^b^	3.21	133 (33.2)	122 (30.4)	146 (36.4)	2.54	0.28
Industrial air pollution^b^	2.09	39 (29.5)	36 (27.3)	57 (43.2)	6.39	0.04

^a^Respondents who rated 4–5 on a 5-point scale, ranging from no exposure (1) to very high exposure (5)

^b^Representing perceived air pollution in the path analysis

**Table 3:** Mean ratings (entire sample) and response frequencies (%) regarding health risk perception from air pollution and worry regarding health risks from air pollution and environmental factors for three groups exposed to different levels of air pollution, and results from group comparisons

	Mean	Exposure level				
How would you rate the risks to your own health caused by the following factors^a^		Low	Medium	High	Chi^2^	*p* value
Traffic exhaust^b^	2.58	86 (33.7)	79 (31.0)	90 (35.3)	0.72	0.69
Street dust^b^	2.44	67 (32.1)	60 (28.7)	82 (39.2)	4.04	0.13
Industrial air pollution^b^	2.29	64 (31.8)	63 (31.3)	74 (36.8)	1.09	0.58
Health risks from air pollution make me worried^c^	3.45	184 (34.9)	173 (32.8)	170 (32.3)	1.29	0.53
How worried are you about health risks from the environment on you and your family?^d^	2.86	105 (36.6)	80 (27.9)	102 (35.5)	4.88	0.09

^a^Respondents who rated 4–5 on a 5-point scale, ranging from no risk (1) to very high risk (5)

^b^Representing perceived air pollution in the path analysis

^c^Respondents who rated 4–5 on a 5-point scale, ranging from strongly disagree (1) to strongly agree (5)

^d^Respondents who rated 4–5 on a 5-point scale, ranging from not at all worried (1) to very worried (5)

**Table 4:** Mean ratings (entire sample) and response frequencies (%) regarding symptoms attributed to environmental factors for three groups exposed to different levels of air pollution, and results from group comparisons

	Mean	Exposure level				
Do the following factors usually cause you some kind of symptoms (e.g. feeling ill, headache, respiratory symptoms)^a^		Low	Medium	High	Chi^2^	*p* value
Traffic exhaust^b^	1.94	31 (29.0)	41(38.3)	35 (32.7)	1.84	0.39
Street dust^b^	2.01	38 (32.2)	37 (31.4)	43 (36.4)	0.43	0.81
Industrial air pollution^b^	1.62	16 (28.6)	18 (32.1)	22 (39.3)	0.97	0.62

^a^Respondents who rated 4–5 on a 5-point scale, ranging from no symptoms (1) to very many symptoms (5)

^b^Representing symptoms in the path analysis

**Table 5:** Response frequencies (%) regarding diseases and self-rated health for three groups exposed to different levels of air pollution, and results from group comparisons

	Exposure level				
	Low	Medium	High	Chi^2^	*p* value
Chronic diseases^a^				4.53	0.34
0	216 (32.9)	214 (32.6)	226 (34.5)		
1	101 (33.2)	108 (35.5)	95 (31.3)		
≥2	17 (42.5)	8 (20.9)	15 (37.5)		
Arthritis^b^	0 (0)	0 (0)	6 (100)	7.95	< 0.001
CVD (any heart disease, high blood pressure or both)^b^	109 (34.9)	105 (33.7)	98 (31.4)	1.02	0.60
Any heart disease	40 (33.3)	38 (31.7)	42 (35.0)	0.18	0.92
High blood pressure	97 (34.4)	94 (33.3)	91 (32.3)	0.32	0.85
COPD^b^	11 (44.0)	5 (20.0)	9 (36.0)	2.2	0.33
Asthma^b^	12 (37.5)	9 (28.1)	11 (34.4)	0.4	0.81
Diabetes^b^	6 (42.9)	5 (35.7)	3 (21.4)	1.04	0.59
Self-rated health^c^ (values 1–2)	159 (32.8)	155 (32.0)	171 (35.3)	1.19	0.55

^a^Compound index of a respondent’s diagnosed diseases including chronic obstructive pulmonary disease (COPD), asthma, cardiovascular disease, diabetes, arthritis

^b^Representing diseases in the path analysis

^c^Respondents who rated 1–2 on a 5-point scale, ranging from very good (1) to very poor (5)

### Path analysis

The hypothesised model was tested with path analysis, which is a form of structural equation modelling for testing and estimating causal relationships using a combination of statistical data and qualitative causal assumptions. This analysis encourages confirmatory rather than exploratory modelling, which means that it starts with a hypothesis (the three factors; air pollution, perceived air pollution and health risk perception that influence health symptoms and diseases), represents it as a model, operationalises the constructs and tests the model. A value for each rated factor in the path analysis and respondent was obtained by averaging ratings across traffic air pollution, street dust and industrial air pollution for a specific factor. We used air pollution data as a continuous variable to exploit its full variability. Root mean squared error of approximation (RMSEA) and Bentler’s comparative fit (CFI) were used as goodness-of-fit indices. An RMSEA value of 0.05 is indicative of a good fit, and a value of 0.08 stands for a reasonable fit (Brown and Cudeck 1993). Divergence from an RMSEA value of 0.05 can be tested, and a nonsignificant *p* value of close fit (PCLOSE) indicates that the RMSEA value is not significantly different from 0.05. A CFI value of 0.95 has been suggested to represent a fairly good fit (Hu and Bentler 1999). To control the model for regional time-invariant confounders (e.g. possible biases arising from differences in socio-economic status, infrastructure) the model was tested in regions with varying degrees of urbanity: in large cities (Tallinn, Tartu, Kohtla-Järve, and Narva regions with mean PM_10_ ${\upmu }\text{g}/$m^3^ of 9.05; SD 4.33), towns (8.52; SD 4.10) and rural areas (9.13; SD 4.50).

## Results

Spearman rho coefficients between the factors included in the path analytic model are given in Table 6. All correlations, except those related to air pollution, were positive and statistically significant. There is a negative, but statistically nonsignificant correlation between air pollution and number of diseases.

**Table 6:** Spearman (rho) correlation coefficients (*p* value) between air pollution, perceived air pollution, health risk perception, symptoms related to these factors, and diseases

	Air pollution (PM_10_, µg/m3)	Perceived pollution	Health risk perception	Symptoms
Perceived pollution^a^	0.042 (0.189)			
Health risk perception^a^	0.027 (0.394)	0.634 (< 0.001)		
Symptoms^a^	0.011 (0.730)	0.569 (< 0.001)	0.689 (< 0.001)	
Diseases	− 0.014 (0.666)	0.066 (0.038)	0.071 (0.025)	0.161 (< 0.001)

^a^Averaged values for traffic air pollution, street dust and industrial air pollution

**Fig. 1:**
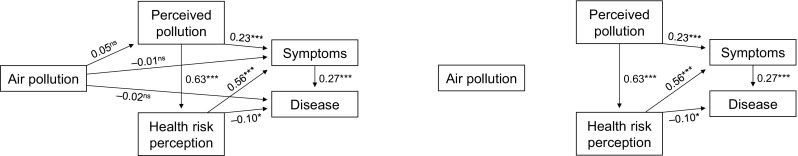
Path analytic model of influences of air pollution, perceived air pollution and health risk perception on symptoms and disease (with and without non-significant paths). Standardised path coefficients are given (*p < 0.05; **p < 0.01; ***p < 0.001)

In testing the theoretical model (Fig. 1, left panel), all path coefficients were significant, except for those coefficients linking air pollution to perceived air pollution, symptoms and disease. The model has good fit (CFI = 1.00; RMSEA = 0.00; PCLOSE = 0.99). Figure 1 (right panel) also shows the model in which the non-significant paths are excluded. This model did not change substantially from the initial, theoretical model and did still indicate good fit (CFI = 1.00; RMSEA = 0.00; PCLOSE = 0.99). Thus, the model in Fig. 1 was considered the final model. Testing of the model in regions with varying degrees of urbanity showed that the model has good fit for three regions (CFI = 0.994; RMSEA = 0.033; AIC = 120.4) (regardless of the level of general pollution). However, the regression coefficients vary between models representing different regions. More explicit differences between regions occur for the relationship between perceived pollution and symptoms; compared to large cities (Regression Weight 0.271; *p* < 0.001) and towns (0.244; *p* < 0.001), this is considerably weaker in rural areas (0.081; *p* = 0.04). As for the relationship between perceived pollution and health risk perception, this is strong in large cities (0.688; *p* < 0.001), but even stronger in towns (0.73; *p* < 0.001), and considerably weaker in the countryside (0.568; *p* < 0.001). The relationship between symptoms and diseases is significant in large cities (0.219; *p* < 0.001), but not in towns and rural areas. Health risk perception and diseases are negatively correlated (0.092; *p* < 0.001) urban areas, yet a small insignificant positive correlation for towns, and an insignificant negative correlation for rural areas.

The negative weak, yet significant (*p* = 0.025) relationship between health risk perception and disease was further explored by post hoc analyses with multiple correlation and multiple regression analysis, testing possible socio-demographic factors explaining this relationship. No distinctive factor, among, for example, sex, age, education, urbanity of the individual’s settlement type, was found to significantly (*α* = 0.05) explain the negative relationship between health risk perception and disease.

## Discussion

The objective of the present study was to test a model of interrelations between physical air pollution, perceived air pollution, health risk perception, symptoms and disease among Estonian inhabitants. Results from the path analysis showed that level of air pollution exposure did not significantly influence perceived pollution and health risk perception, symptoms or diseases. Perceived exposure was found to influence health risk perception, which, in turn, influenced health symptoms and diseases. Furthermore, perceived exposure was found to influence symptoms, which, in turn, influenced diseases. The model fitted equally well for regions with varying level of urbanity. However, the regression coefficients for the different regions show that compared to rural areas, in large cities and towns, the perceived pollution is more likely to be associated with symptoms, and symptoms are associated with diseases only in large cities. The relationship between perceived pollution and perceived risk is strong in large cities, but even stronger in towns, and considerably weaker in the countryside. People more likely recognise pollution and its impacts on personal health when operating in urban areas, independently of the pollution level in their specific living area. Post hoc analysis shows that this heightened perception of pollution and symptoms that may lead to disease can be explained by higher level of education and related attention to health effects in cities. In large cities, chronic diseases are also more frequent, which might sensitise people to physical stress in cities.

Caution should be taken regarding the direction of effects in the proposed model. Path analysis does not test the cause–effect direction between factors per se. For example, it cannot be excluded that perceived pollution and health risk perception or disease and health risk perception mutually affect each other. It is difficult to distinguish the impact on health due to air pollution from the impact of, for example, noise (Lex Brown and van Kamp 2017) and lack of recreational areas in dense urban areas (Lee and Maheswaran 2011). Thus, we cannot exclude that other effects may amplify the perceived risk and symptoms attributed to air pollution and health conditions. Explanations for the nonsignificant effect of air pollution on the other factors in the model can be the relatively low levels of air pollution (Air Quality Directive 2008/50/EC requirements are fulfilled in Estonia) as well as relatively large grid cell of the air pollution dispersion model, by which the urban gradient might have been partly hidden. For instance, Miller et al. (2007) have shown lower hazard ratios for between-city exposure data compared to within-city data. Furthermore, Punger and West (2013) assessed fine particle health effects with different dispersion model resolutions and found smaller effects with models with larger grid cells. For a recent discussion on how spatial and temporal aggregation may mask a heterogeneity in air pollution and its health effects, see, e.g. Gehrsitz (2017).

The results are likely to have been affected by the rather low levels of air pollution, which are also reflected by the low ratings of perceived air pollution [range 2.09–3.19 on a scale ranging from no exposure (1) to high exposure (5)], health risk perception (2.29–3.45) and symptoms (1.62–2.01).

The analysis showed no significant income differences between exposure groups. Therefore, a self-selection thesis of socio-economically less secured people staying in environmentally degraded areas and more well-off people being able to stay closer to greener areas (see e.g. Bilger and Carrieri 2015) was not supported by the present analysis based on data from Estonia with relatively low average levels of air pollution.

A direct effect of exposure on perceived pollution, health risk perception, symptoms or diseases may have been prevalent if higher levels of air pollution were present. For instance, Cesaroni et al. (2008) observed a much higher correlation between modelled air pollution and self-reported traffic intensity in Rome compared to the RHINE cohort which was exposed to significantly lower concentrations (Krage Carlsen et al. 2016). Nevertheless, earlier epidemiological studies in Estonia have shown PM_10_ affecting health due to both short-term (e.g. Läll et al. 2013) and long-term (e.g. Orru et al. 2009; Pindus et al. 2016) exposures, as well as affecting life satisfaction (Orru et al. 2016), despite relatively low levels of air pollution with rare exceedances of limit values during last years (http://ohuseire.ee/en). Post hoc analyses did not identify a single explanatory socio-demographic factor for the negative association between health risk perception and diseases. Yet, it is possible that a combination of such factors may contribute to the association. For example, health risk perception was highest among individuals aged 30–44 years, whereas their level of disease was relatively low compared to other age groups. It is possible that people with higher perceived risk may be more likely to engage in health protection that leads to better health outcomes (Ferrer and Klein 2013).

PM_10_ can be considered a good indicator for the items used as proxies for perception of air pollution (traffic exhaust, street dust and industrial air pollution) in this analysis. However, the reported perception of air pollution in surveys may also include other perceived pollutants such as H_2_S, phenols and benzene that occur particularly in eastern, industrialised parts of Estonia (Orru et al. 2016). Thus, a lack of link between PM_10_ and perceived air pollution may also be caused by the fact that other pollutants may trigger perceived exposure to air pollution.

The arthritis cases (*n* = 6) were significantly more frequent in high-exposure groups. This suggests that air pollution and other environmental stressors may increase the risk of inflammatory conditions that lead to, for example, arthritis. However, the small number of cases calls for caution in the interpretation.

The findings suggest that perceived pollution and health risk perception play significant roles in understanding and predicting environmentally induced symptoms and chronic diseases. Perceived air pollution may have resulted in negative affect, stress-induced physiological activity and thereby health symptoms. Health risk perception involves a top-down, defence mechanism that may influence the occurrence of symptoms and diseases. The individuals’ beliefs about and attitudes toward the air pollution provoke responses to the exposure. If the individual believes that the exposure is hazardous and/or has negative attitudes toward the exposure, this will result in symptoms and disease that may guide an individual to avoid the exposure. The impact of health risk perception on health has been demonstrated in several well-controlled experimental studies (e.g. Andersson et al. 2013; Crichton and Petrie 2015).

The results of this study are of interest in relation to the study by Stenlund and associates (2009) in which health effects of exposure to dust and soot pollution in a residential sample were investigated, using a procedure very similar to that used in the present study. Based on Stenlund et al. (2009) analysis, annoyance, but not health symptoms, was found to be directly triggered by air pollution, whereas the relationship between air pollution and symptoms was mediated by perceived pollution and health risk perception. In a later study, Claeson and colleagues (2013) found the impact of odorous air pollution on annoyance and symptoms to be mediated by perceived pollution and health risk perception. An interpretation of the results from these two studies and the current investigation is that perceived pollution and health risk perception may play a stronger role for the development of health symptoms when the exposure is attributed to one concrete point source (in Stenlund et al. 2009, steel industry in Oxelösund) or when odorous sources are considered (in Claeson et al. 2013, biofuel refinery in Värnamo) compared to when average mean annual exposure levels are considered on a territory of Estonia. However, a significant association between exposure and perceived industrial pollution indicates that the effect of exposure on perceived pollution, health risk perception, symptoms or diseases could appear in the case of industrial pollution. As pointed out by Geelen et al. (2013), attitudes to risk source matter: compared to traffic exhausts and more seasonal street dust, industry pollution is considered less controllable by individuals and thus worrying. Therefore, they might be more attentive to potential levels of industrial pollution, which increases the reporting of being exposed to industrial air pollution. People being more attentive to industrial air pollution compared to other air pollution sources with an impact on perceived pollution and symptoms should be explored in further studies.

In conclusion, the path analyses suggest that perceived air pollution and health risk perception play very important roles in understanding and predicting health symptoms and diseases in environments with relatively low annual mean levels of air pollution. Air pollution levels even below official safety levels may still pose serious health threats, especially if they are recognised and perceived as unhealthy by inhabitants. One important message is that care should be taken while informing people about the health effects of the exposure, since health risk perception may induce additional health consequences.
